# CD8^+^ Treg Cells Associated with Decreasing Disease Activity after Intravenous Methylprednisolone Pulse Therapy in Lupus Nephritis with Heavy Proteinuria

**DOI:** 10.1371/journal.pone.0081344

**Published:** 2014-01-27

**Authors:** Yi-Giien Tsai, Chia-Ying Lee, Tze-Yi Lin, Ching-Yuang Lin

**Affiliations:** 1 Departments of Pediatrics, Changhua Christian Hospital, Changhua, Taiwan; 2 School of Medicine, Chung Shan Medical University, Taichung, Taiwan; 3 Division of Pediatric Nephrology, China Medical University Hospital, Taichung, Taiwan; 4 Department of Pathology, China Medical University Hospital, Taichung, Taiwan; 5 Clinical Immunology Center, China Medical University Hospital, Taichung, Taiwan; 6 College of Medicine, China Medical University, Taichung, Taiwan; Northwestern University Feinberg School of Medicine, United States

## Abstract

We focus on the role of CD8^+^ Treg cell in Intravenous methyl-prednisolone (IVMP) pulse therapy in forty patients with active Class III/IV childhood lupus nephritis (LN) with heavy proteinuria. IVMP therapy for five days. From peripheral blood mononuclear cells (PBMCs) and renal tissues, we saw IVMP therapy definitely restoring both CD4^+^CD25^+^FoxP3^+^ and CD8^+^CD25^+^Foxp3^+^ Treg cell number plus greater expression with intracellular IL-10 and granzyme B in CD8^+^FoxP3^+^ Treg from PBMCs. IVMP-treated CD8^+^CD25^+^ Treg cells directly suppressed CD4^+^ T proliferation and induced CD4^+^CD45RO^+^ apoptosis. Histologically, CD4^+^FoxP3^+^ as well as CD8^+^FoxP3^+^ Treg cells appeared in renal tissue of LN patients before IVMP by double immunohistochemical stain. CD8^+^FoxP3^+^ Treg cells increased in 10 follow-up renal biopsy specimens after IVMP. Reverse correlation of serum anti-C1q antibody and FoxP3^+^ Treg cells in PBMNCs (r = −0.714, P<0.01). After IVMP, serum anti-C1q antibody decrease accompanied increase of CD4^+^FoxP3^+^ Treg cells. CD8^+^Treg cells reduced interferon-r response in PBMCs to major peptide autoepitopes from nucleosomes after IVMP therapy; siRNA of FoxP3 suppressed granzyme B expression while decreasing CD8^+^CD25^+^Treg-induced CD4^+^CD45RO^+^ apoptosis. Renal activity of LN by SLEDAI-2k in childhood LN was significantly higher than two weeks after IVMP (P<0.01). CD8^+^FoxP3^+^ Treg cells return in post-IVMP therapy and exert crucial immune modulatory effect to control autoimmune response in LN.

**Trial Registration:**

**DMR97-IRB-259**

## Introduction

Childhood lupus nephritis (LN) remains a significant therapeutic challenge due to its complex etiopathogenesis and unpredictable course. Systemic lupus erythematosus (SLE) is characterized by autoantigen-deriven interactions between autoreactive Th and B cells, spawning production of somatically mutated IgG autoautibodies against apoptotic nuclear antigens (Ags) [1], [2], pathogenic IgG autoantibodies belonging to Th1- or interferon gamma (IFNr)-dependent subclass contributing differentiation of autoimmune Th cell with concomitant decrease in regulatory T (Treg) cells [3]. Although immunological defect of SLE is complex. Treg cells play a vital role in autoreactive cell expansion [3]. CD4^+^CD25^+^ Treg cells have potent immunosuppressive function and contribute to immunological self-tolerance in SLE [4], [5]. Recent studies demonstrate Treg cell number as inversely correlated with disease activity, a mechanism that may benefit treatment of LN [4], [5].

CD8^+^ T cells are also abnormal in SLE patients, less competent in cytotoxic activity [6]. CD8^+^ Treg cells expressing transcription factor Foxp3 with regulatory function in maintaining self-tolerance have recently been identified [7]. CD8^+^CD25^+^FoxP3^+^ T cells can be generated by continuous antigen (Ag) stimulation [7], [8]. CD8^+^Treg cells were first identified in human tonsils; upon *in vitro* activation, FoxP3^+^CD8^+^Treg cells were shown to inhibit T cell proliferation directly [8]. CD8^+^Treg cells seem to perform a regulatory function to limit autoimmune disease in experimental models [7]–[12]. Human CD8^+^ Treg cells are implicated in autoimmune disorders: e.g., multiple sclerosis, inflammatory bowel disease [13]. Suppressive CD8^+^Foxp3^+^ Treg cells appear after T cell receptor stimulation, suppressing cellular proliferation of CD4^+^ naïve and effector T cells via cell-cell contact lysis or soluble factors like IL-10 and TGF-β [7], [14]–[15]. Systemic immunization with allergen in mice induces CD8^+^ Treg cells to inhibit allergic diarrhea, suggesting their pivotal role in limiting autoimmune disease [16].

CD8^+^CD25^+^ Treg cells have suppressive ability typically associated with CD4^+^ Treg [17]–[21]. Interaction between subsets of Treg cells that protect against autoimmune diseases remains unclear. Foxp3-expressing CD8^+^ T proved vital for CD4^+^CD25^+^ Treg cells induced by a tolerogenic peptide to suppress murine lupus [22]. Animal models of SLE suggest defective CD8^+^ Treg cells associated with LN [23] and induction of CD8^+^ Treg cells with immune tolerance of lupus mice [24]. Complement activation enhance leukocyte infiltration and production of pro-inflammatory cytokines in the kidney [25]. Active LN in children always had high level of complement activation [26], [27]. Clinically, kidney involvement in LN may vary from mild hematuria or proteinuria to acute or chronic kidney disease. Renal pathology can have a broad range of Class I–VI. Class III and IV both were diffuse proliferative glomerulonephritis [28].

While conventional treatment with intravenous methylprednisolone (IVMP) suppresses disease activity and complement activation in children with LN, some patients still develop progressive renal injury; some who respond to treatment remain at risk of relapse [29]. Yet no study rates IVMP effect on Treg cells to maintain immune tolerance from active Class III and IV LN. This study focuses on the role of CD8^+^ Treg cells in IVMP therapy, 40 LN patients receiving IVMP and 10 historical control patients only treated with oral prednisolone. Our results provide evidence that CD8^+^ Treg cells play an important role of inducing treatment immune modulation by IVMP.

## Results

### Clinical response of LN patients after IVMP pulse therapy

Median duration between LN diagnosis and time of study was 314 (range: 0 = 1148) days. LN was the first manifestation in 37 of 40 patients; for the rest, thrombocytopenia and anemia were the first manifestations of SLE. Descriptors of SLEDA present at the time of blood withdrawal before IVMP in study population included arthritis, hematuria, proteinuria, new molar rash, low complement, mucosal ulcer, increased DNA binding, hemolytic anemia, thrombocytopenia and leucopenia (Table 1). There was a sharp rise of SLEDAI score two weeks after IVMP treatment (Table 2). At study entry, mean serum C3 level was 63.5±21.1 mg/dl, C4 level 11.1±6.7 mg/dl and anti-dsDNAAb was 258.1±42.2 U/ml. Increase in both C3 and C4, decrease in anti-dsDNA Ab with daily urine protein loss and activity of LN by SLEDAI-2k in childhood LN before IVMP were significantly higher than those two weeks after IVMP (Table 2). All patients showed improved two weeks after IVMP pulse therapy.

**Table 1:** Baseline characteristics.

Cases	IVMP (n = 40)	controls (n = 10)
Age (years)	15.2±3.2	20.4±4.3
Body weight(kg)	52.3±7.5	53.4±8.3
Disease duration (years)	0.7±0.2	
F∶M	32∶8	8∶2
Renal histology		
Class III	18 (45%)	0
Class IV	22(55%)	0
ARB/ACEI	34(85%)	0
Urinary preotein (mg/d)	1996.3±580.1	0
Serum Cr (mg/dl)	0.61±0.2	0.57±0.2
GFR (ml/min/1.73 m^2^)	118.9±9.4	106.8±7.2

Mean±SD reported for quantitative variables, F: female, M: Male. Cr:creatinine, angiotensin-converting enzyme inhibitors (ACEIs), angiotensin-receptor blocker (ARBs).

**Table 2:** Disease activity and laboratory variables in both groups before and two weeks after IVMP treatment.

	IVMP	
Cases	Pre-IVMP	Post-IVMP
C3(mg/dl)	63.5±21.1	90.3±20.3*
C4(mg/dl)	11.1±6.7	15.5±5.3**
Anti-dsDNA Ab(U/ml)	258.1±42.2	162.8±31.6*
Daily urine protein(mg)	1996.3±580.1	723.9±289.3*
SLEDAI score	7.2±3.8	5.2±1.4**
SLEDAI-2k median (range)	16(9–24)	4(2–8)*

* P<0.01,

** P<0.05 compared to pre-IVMP pulse therapy.

### Increase of CD4^+^CD25^+^FoxP3^+^ and CD8^+^CD25^+^FoxP3^+^ Treg cells following IVMP pulse therapy

**Figure 1:**
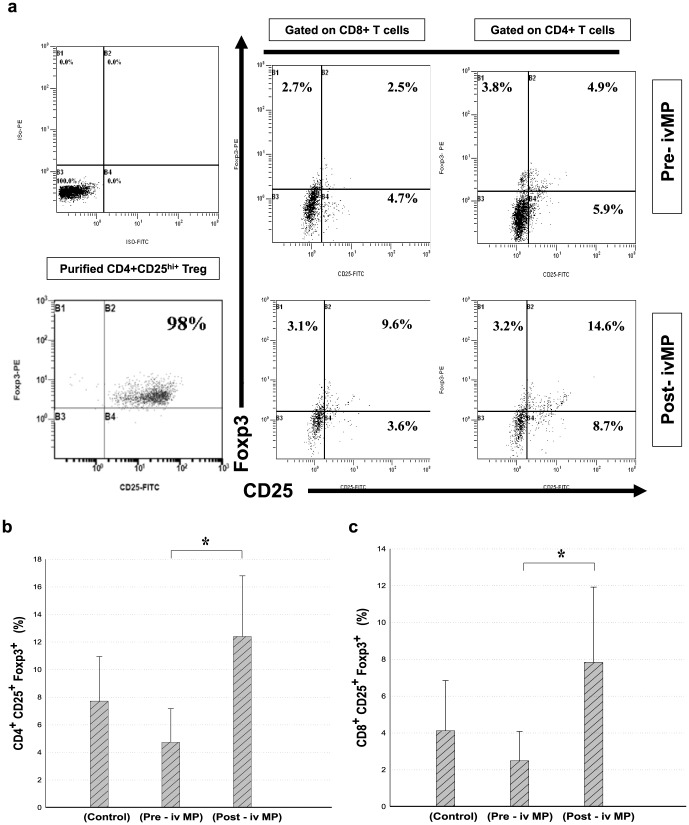
CD4^+^CD25^+^FoxP3^+^ and CD8^+^CD25^+^FoxP3^+^ Treg cells before and after IVMP pulse therapy. (a) PBMCs from LN patients during IVMP pulse therapy were stimulated with anti-CD3 mAb stimulation (5 ug/ml) and IL-2 (10 U/ml) for five days, and cells with intracellular expression of FoxP3 were analyzed for CD4^+^CD25^+^ and CD8^+^CD25^+^ T cells, representative figures shown. Analysis of CD4^+^CD25^+^FoxP3 Treg cells (b) and CD8^+^CD25^+^FoxP3 Treg cells (c) in PBMCs before and after IVMP pulse therapy by flow cytometry. Bars represent mean ± SD.

Both CD4^+^CD25^+^FoxP3^+^ and CD8^+^CD25^+^FoxP3^+^ Treg cells were analyzed by flow cytometry (Fig. 1a) for surface markers and intracellular FoxP3 expression simultaneously in PBMCs following anti-CD3 mAb stimulation **(5 µg/ml) and IL-2 (10 U/ml)** **for five days** in each group. As shown in Table 3 **and** **Figure 1**, on Day 6 after IVMP therapy, both CD4^+^CD25^+^FoxP3^+^ and CD8^+^CD25^+^FoxP3^+^ Treg cells increased in number. There was no correlation between increasing serum C3, C4 levels decreasing anti-dsDNA level and daily urinary protein loss.

**Table 3:** CD4^+^CD25^+^FoxP3^+^ and CD8^+^CD25^+^FoxP3^+^ Treg cell numbers following anti-CD3 mAb stimulated PBMCs in both groups before (Day 0) and after IVMP treatment (Day 6).

	IVMP(n = 40)		
Cases	Pre-IVMP	Post-IVMP	Control(N = 10)
CD4^+^CD25^+^FoxP3^+^ Treg	4.37±2.43	12.37±4.34*	7.72±2.41*
CD8^+^CD25^+^FoxP3^+^ Treg	2.49±1.59	7.84±4.08*	4.05±2.42*

* P<0.01 vs. pre-IVMP, data shown as mean±SEM.

### Reverse correlation of serum anti-C1q antibody and CD8^+^FoxP3^+^ Treg cells

Children with active LN showed markedly higher concentration of anti-C1q antibody (842.5±72.6 U/ml) before than after IVMP therapy (157.4±26.3 U/ml, P<0.0001). Before IVMP, children with Class III and IV LN manifested significant inverse correlation between level of anti-C1q antibody and CD8+FoxP3+Treg cells in PBMCs (r = −0.714, P<0.01). After IVMP therapy, serum anti-C1q antibody decreased, accompanied by increasing CD8^+^FoxP3^+^ Treg cells.

### Increase of CD8+FoxP3+ Treg cells correlated with renal response to IVMP therapy

**Figure 2:**
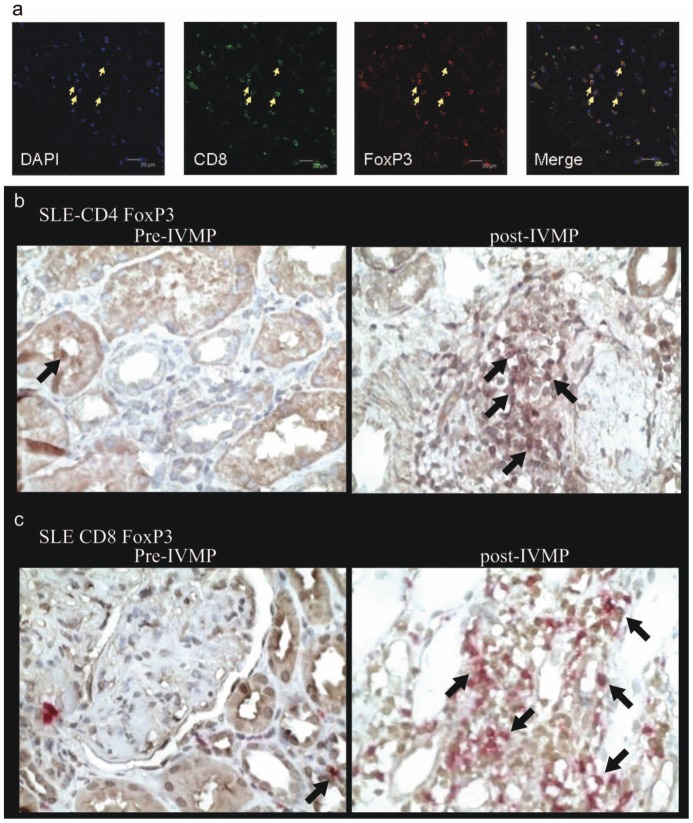
Confocal microscopic analysis of renal biopsy in active Class IV LN patient before IVMP pulse therapy. (a) Specimen was stained for CD8, FoxP3, and 4′, 6-diamidino-2-phenylindole (DAPI) (nuclear stain). White arrows indicate CD8^+^FoxP3^+^ cells. (b) (c) right CD4^+^FoxP3^+^ and CD8^+^FoxP3^+^Treg cell expression significantly decreased before IVMP pulse therapy in renal tissue of Class IV LN (n = 50) FoxP3^+^ (brown); CD4^+^ or CD8^+^ (pink). (b) (c) left CD4^+^FoxP3^+^ and CD8^+^FoxP3^+^ Treg cell expression significantly increased after IVMP pulse therapy in renal tissue of follow-up biopsies in Fig. 2a patient.

Renal histological sections revealed endoproliferative change and glomerulonephritis with inflammatory cell infiltration and expansion of the mesangial matrix, as well as marked mesangial proliferation in active class III/IV LN before IVMP therapy. Lymphocyte subpopulations were mainly present in the renal interstitium; sometimes cells were found in the glomeruli, vessels and tubuli with tubulitis. Confocal microscopic analysis of renal biopsy specimen before IVMP pulse therapy revealed CD8^+^FoxP3^+^ Treg cells present in active LN patients (Fig. 2a). CD4^+^FoxP3^+^ and CD8^+^FoxP3^+^ Tregs were also studied in renal biopsy specimens from active LN cases before IVMP therapy by microscopic analysis using double immunohistochemical stain with CD4 or CD8 and FoxP3. Results showed a few of, both CD4^+^FoxP3^+^ and CD8^+^FoxP3^+^ Treg cells (Fig. 2b, 2c, **Table 4**) and absence of CD8^+^ granzyme B^+^ cells before IVMP therapy. Ten cases received follow-up renal biopsy. Repeat histological sections revealed improvement: markedly less CD3^+^ interstitial lymphocyte infiltration but definitely more CD4^+^FoxP3^+^ and CD8^+^FoxP3^+^ Treg and CD8^+^ granzyme B^+^ cells after IVMP therapy (**Table 4**). There was no correlation between either CD4^+^FoxP3^+^ or CD8^+^FoxP3^+^Treg cells in renal tissue and eGFR or chornic index or daily urine protein or serum C3,C4, anti-dsDNA Ab levels. Histopathological score of mesangial proliferation, as well as sclerosis score before and after IVMP pulse therapy, showed proliferation score sharply decreased (1.72±0.48 vs 0.82±0.51,P<0.05), as did sclerosis score (2.17±0.24 vs 1.42±0.34,P<0.05).

**Table 4:** Number of interstitial CD3^+^, FoxP3^+^, CD4FoxP3^+^ and CD8^+^FoxP3^+^ cells in renal biopsy.

infiltrating cell type	SLE, IVMP		Controls (n = 6)
	Before (n = 40)	After (n = 10)	
interstitial CD3^+^ cells	27.2±6.4	29.4±4.7	5.1±1.3
interstitial FoxP3^+^ cells	1.4±0.8*	12.1±3.8	2.2±0.7
interstitial CD4^+^FoxP3^+^ cells	0.6±0.4*	3.2±0.8*	1.1±0.5
interstitial CD8^+^FoxP3^+^ cells	0.8±0.4*	8.4±3.4*	0.6±0.7

* P<0.05 vs CD3+ cell group, data shown as mean±SEM.

### IVMP therapy increased CD8^+^CD25^+^ Treg cells expressing granzyme B and IL-10

**Figure 3:**
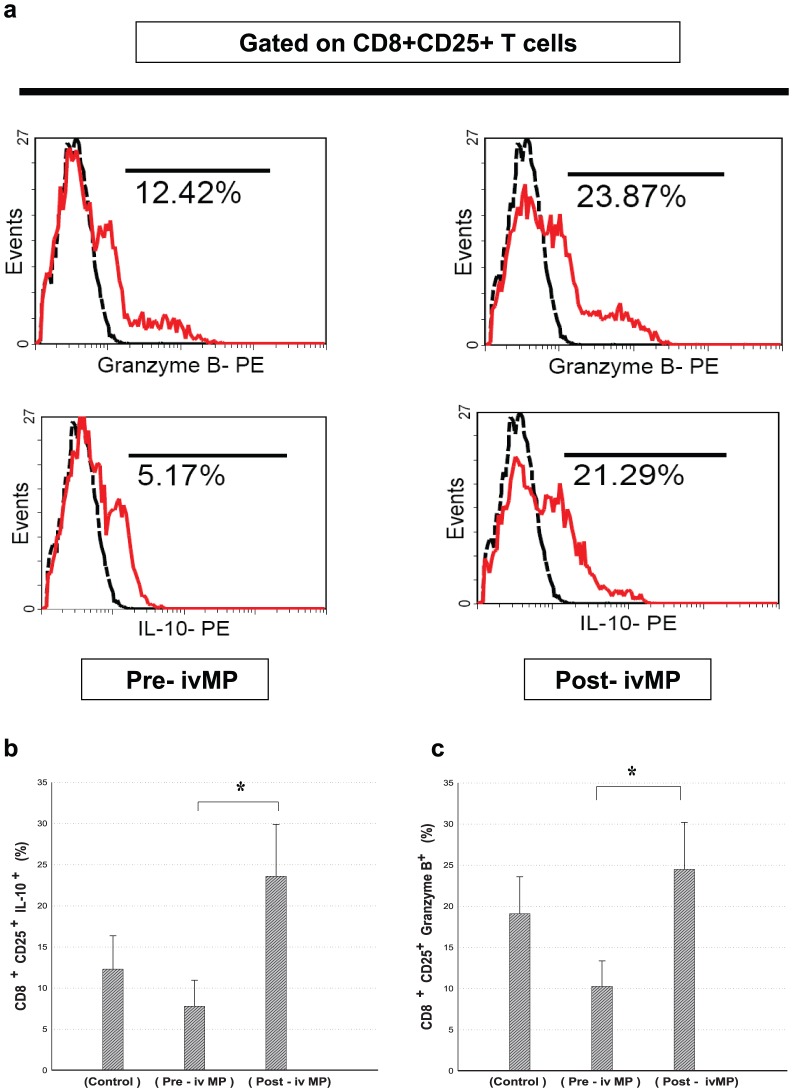
Intracellular IL-10 and granzyme B levels in CD8^+^CD25^+^ Treg cells before and after IVMP. PBMCs were stimulated with anti-CD3 mAb for five days, followed by stimulation of PMA (10 ng/ml) plus ionomycin (1 µg/ml) for the last five hours, with addition of brefeldin A (10 µg/ml) for the final hour. Intracellular expression of IL-10 and granzyme B was measured by gating in CD8^+^CD25^+^ T cells, using flow cytometry. Isotype control (dotted line). (a) Results of 30 paired experiments for IL-10 (b) and granzyme B (c) production by PBMCs (* *p*<0.05).

To ascertain whether increasing CD8^+^CD25^+^ Treg cells correlated with IL-10 and granzyme B expression, anti-CD3 mAb stimulated PBMCs were activated for five hours with PMA and ionomycin, then stained for intracellular IL-10 and granzyme B to characterize expression in CD8^+^CD25^+^ T cells (Fig. 3a). Comparing CD8^+^CD25^+^IL-10^+^ and granzyme B expressing CD8^+^CD25^+^ T cell numbers from PBMCs of LN patients before and after IVMP pulse therapy showed CD8^+^CD25^+^IL-10^+^ T cells significantly higher (7.88±3.16 vs 23.60±6.3970, P<0.05)(Fig. 3b), as did granzyme B expressing CD8^+^CD25^+^ T cells (10.28±3.10 vs 24.59±5.7570, P<0.05)(Fig. 3c).

### IVMP therapy restored suppressive activity in CD8^+^ Treg cells of LN patients

**Figure 4:**
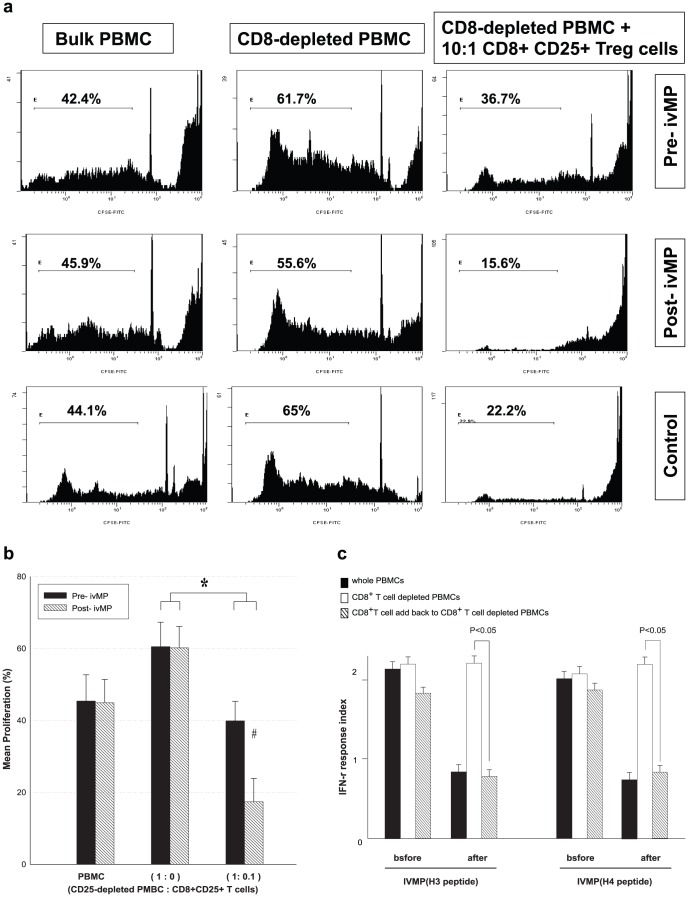
CD4^+^ cell proliferation in the presence of CD8^+^CD25^+^ Treg cells during IVMP. (a) CFSE-labeled cells (Bulk PBMCs and CD8^+^-depleted PBMCs) were pretreated with anti-CD3 mAb for five days, CD8^+^-depleted PBMCs incubated with purified CD8^+^CD25^+^ T cells at a ratio of 10:1, proliferation of CD4^+^ T cells analyzed by flow cytometry. (b) There was significant suppression (*) of CD^+^ cells proliferation in the presence of CD8^+^CD25^+^ regulatory T cells compared to CD8^+^ depleted PMNCs alone. There was significant suppression (^#^) of CD4^+^ T cell proliferation after IVMP during SLE, data calculated from 20 paired experiments. (*^#^ indicates *p*<0.05). (c) Th1 type IFN-r response to critical peptide epitopes (H3: 115–135, H4: 16–39) in PBMCs of LN patients before and after IVMP pulse therapy. CD8^+^ T cells significantly suppressed IFN-r response after IVMP pulse therapy. Data were calculated from 20 paired experiments; bars represent mean ± SD.

To determine whether CD8^+^CD25^+^ Treg cells regulate CD4^+^ T cell proliferation, PBMCs and CD8^+^-depleted PBMCs from LN patients from Pre-IVMP status and controls were stimulated with anti-CD3 mAb and then labeled with CFSE. Purified, non-CFSE labeled CD8^+^CD25^+^ Treg cells were added to CD8^+^-depleted PBMCs and cell proliferation measured. In a representative study, CD4^+^ T cells proliferation induced by anti-CD3 was enhanced following CD25^+^ T cell depletion; addition of CD8^+^CD25^+^ Treg cells significantly inhibited CD4^+^ T cells proliferation, with no commensurate rise in proportion of CFSE undiluted cells, suggesting cytotoxic effect of CD8+CD25+ Treg cells (Fig. 4a). Data from 20 lupus patients show CD8^+^CD25^+^ Treg cells less suppressed CD4^+^ T cell proliferation in active LN, IVMP increased CD8^+^CD25^+^ Treg cells suppression of CD4^+^ T cells proliferation (Fig. 4b).

### Increasing CD8^+^CD25^+^ Tregs induce CD4^+^CD45RO^+^ apoptosis in LN cases after IVMP therapy

**Figure 5:**
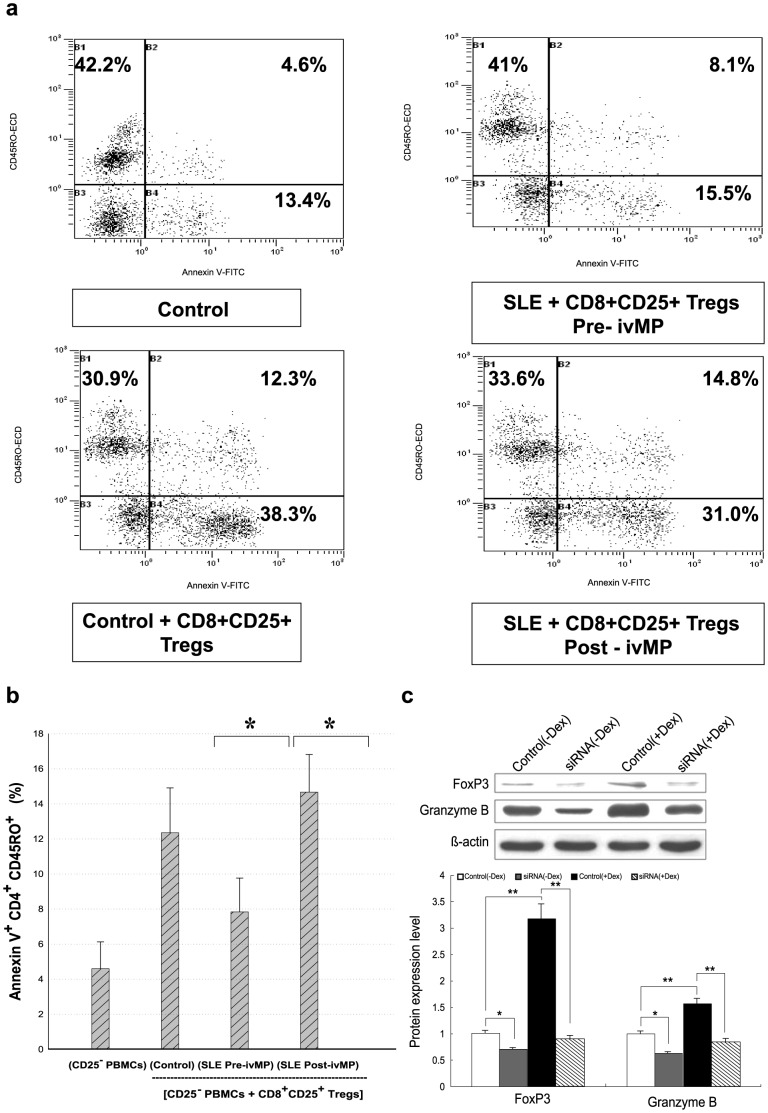
Double fluorescence study by flow cytometry of CD45RO lymphocytes subpopulations and apoptosis by CD8^+^CD25^+^ regulatory T cells during IVMP. (a). CD25^+^-depleted PBMCs were co-cultured with CD8^+^CD25^+^ Treg cells from control subjects or CD8^+^CD25^+^ Treg cells after IVMP during SLE. Apoptosis was simultaneously determined by Annexin V labeling and negative PI gating, representative histograms shown. (b). Percentage of Annexin V-positive CD4^+^CD45RO^+^ cells rose sharply after addition of Treg cells during IVMP, data calculated from 20 paired experiments. (*^#^ *p*<0.05). (c). siRNA of FoxP3 decreased granzyme B protein expression in CD8^+^CD25^+^ Treg cells if pretreated with nucleosomal histone peptide epitope (H3:115–135) and dexamethasme (50 nM). Right column shows control without dexamethasone, nucleosomal histone peptide epitope and siRNA treatment; left column second control pretreated with nucleosomal histone peptide epitope with IL-2 (10 U/ml) for 3 days and third day treated with dexamethasone for 24 hours. Middle left column was a control RNA transfection for FoxP3 siRNA. Middle right column was pretreated with nucleosomal histone peptide for 3 days and third day treated with dexamethasone for 24 hours their FoxP3 siRNA treatment for 48 hours. Data were derived from three independent experiments; bars represent mean±SD.

Our study probed CD8^+^CD25^+^ Treg cells triggering CD4^+^CD45RO^+^ apoptosis by IVMP pulse therapy. Effect of CD8^+^CD25^+^ Tregs on CD4^+^CD45RO^+^ apoptosis was rated by percentage of Annexin V-positive CD4^+^CD45RO^+^ T cells in CD25^+^-depleted PBMCs from Pre-IVMP status cocultured with purified CD8^+^CD25^+^ Treg cells (Fig. 5a). Ratio of Annexin V-positive CD4^+^CD45RO^+^ T cells rose after addition of CD8^+^CD25^+^ Treg cells to CD25^+^-depleted PBMCs from LN patients (Fig. 5b).

### CD8^+^CD25^+^Treg cells reduced IFN-r response in PBMCs to major peptide autoepitopes from nucleosomes of LN patients after IVMP therapy

To ascertain whether interferon gamma (IFN-r) response of CD4^+^ T cells decreasing response to autoantigens after IVMP pulse therapy, results showed IFN-r response of CD4^+^ T cells to autoantigen peptide epitopes (H3: 115–135; H4: 16–39) increasing in CD8^+^CD25^+^Tregs' depleted condition. With CD8^+^CD25^+^Treg cells added back to CD8^+^ CD25^+^Treg cells depleted PBMCs from LN patients after IVMP, IFN-r response of CD4^+^ T cells was suppressed (Fig. 5c). This suppression of CD8^+^CD25^+^Treg in IFN-r response to CD4^+^ T cells was not found before IVMP pulse therapy.

### siRNA of FoxP3 suppress granzyme B expression

We further analyzed contribution of FoxP3 to increasing granzyme B protein expression induced by CD8^+^CD25^+^Treg proliferation and FoxP3 synthesis by small interfering RNA (siRNA)-mediated knockdown experiments. As of Day 5, FoxP3 knockdown showed suppressive effect on decreasing granzyme B protein expression induced by CD8^+^CD25^+^Treg cells from ΔOD 0.32±0.4 to 0.12±0.2 (P<0.01) (Fig. 5c) and decreasing CD4^+^CD45 RO^+^ cell apoptosis (14.2±0.3 to 6.9±0.4%, p<0.001).

## Discussion

There exist several hypotheses for the IVMP therapy mechanism: (1) immune ablation eliminates autoreactive T cell clone; (2) autoreactive T cell clones are rendered tolerant; (3) regulatory networks control autoreactive T cells and complement activation. In fact, upon immune reconstitution, default mechanism of the immune system is self-tolerance; Treg function plays a crucial role. Most studies of Treg cells in human SLE focus on CD4^+^ Treg, with results conflicting [30] but suppressive function of CD4^+^Treg cells impaired [31]. Recent evidence shows corticosteroids influencing regulatory cell population or even generating Treg cells [32]. Besides SLE patients, treatment with glucocorticoids yields increase in Treg cells and FoxP3 levels in patients with asthma [33], immune thrombocytopenia purpura patients [34] and multiple sclerosis [35].

Observation in murine SLE indicates CD8^+^Tregs playing a lead role in disease activity [36]. We found significantly low number of both CD4^+^FoxP3^+^ and CD8^+^FoxP3^+^ Tregs in pre-IVMP therapy; frequency of these subsets after IVMP was restored. We also analyzed suppressive function of CD4^+^FoxP3^+^Treg cells after IVMP therapy, but those had minor suppressive function. In our study, IVMP increased CD8^+^CD28^+^ Treg cells number and expressing granzyme B and IL-10, restored strong suppressive activity on CD4^+^ T proliferation, increasing CD8^+^CD25^+^ Tregs induced CD4^+^CD45^+^RO^+^ apoptosis, reducing IFN-r response in PBMCs to major peptide autoepitopes from nucleosomes. Increasing CD8^+^ Treg cells *in vivo* correlated with renal histopathological improvement and anti-C1q antibody response. Another study found LN patients in remission, with conventional steroid-induced remission exhibiting no suppressive function on CD4^+^ T proliferation [37]. These indicate a different mechanism in IVMP therapy to obtain clinic efficacy; understanding those mechanisms may enhance LN therapy. Table 2 shows most post-IVMP LN patients improving SLEDAI score. CD8^+^ Treg cells from these patients showed strong suppressive function both *in vitro* and *in vivo*. Auto-antibodies against C1q are directed against a highly functional molecule with vital roles in autoimmunity. We proved strong correlation between occurrence of anti-C1q antibodies in Class III/IV LN. Increasing evidence shows inactive classic complement pathway definitely improved in Class III/IV LN, and CD8^+^ Treg cells seem to play a suppressing complement activation in classic pathway.

Earlier studies showed up-regulation of Th1 cytokine IL-2 and IFN-γ together with decreased production of Th2 cytokine IL-4 up-regulating autoantibody production by B cells and associated with disease activity [38]. Administration of anti-IFN-γ antibodies [39] or IL4pDNA [31] delays lupus development in NZB/W F1 mice. Horwitz et al. reported [a] depleting CD8^+^ T cells from active lupus PBMCs can reduce polyclonal IgG production and [b] adding autologous CD8^+^ T cells back to PBMCs can reconstitute this Ab production [5]. Prior studies show, in contrast to normal T cells, SLE patients' T cell response to nucleosomal histone peptide epitopes by producing intracellular IFN-r and inducing anti-DNA autoantibodies [40]. Our study proves, before IVMP pulse therapy, poor CD8^+^CD25^+^Treg suppressive effect on IFN-r production response of CD4^+^ T cell in PBMC to nucleosomal autoepitopes. This indicates functional defect in CD8^+^Treg cells in active LN patients. After IVMP pulse therapy, IFN-r production response of CD4^+^ T cells in PBMC was suppressed by CD8^+^CD25^+^Treg cells. Overall observations indicate IVMP pulse therapy playing an important role in restoring CD8^+^ CD25^+^ Treg cells induced self-tolerance in post IVMP pulse therapy of LN cases.

Post-IVMP CD8^+^Treg cells strongly express IL-10, and granzyme B accompanies CD8^+^Treg cell suppression, supporting a key role of increasing systemic CD8^+^CD25^+^Treg cells after IVMP pulse therapy to suppress T cell proliferation and increasing CD4^+^CD45RO^+^ apoptosis. In renal tissue, during active lupus, increasing CD8^+^FoxP3^+^ T cells may inhibit T proliferation by cell-cell contact [41] and increase production of suppressive cytokine IL-10. Source of increased CD8^+^FoxP3^+^Treg cells in renal tissue during IVMP pulse therapy may migrate from systemic CD8^+^FoxP3^+^Treg cells to limit local inflammation. Taken together, results suggest IVMP pulse therapy playing a key role in redistribution of CD8^+^FoxP3^+^Tregs and limiting autoreactive T cell mediated autoimmune response by increasing IL-10 and granzyme B production. CD4^+^CD45RO^+^ T apoptosis during IVMP pulse therapy represents a unique down-regulatory mechanism to prevent continuous activation of autoimmune response by autoantigen. We proved that CD8^+^ CD25^+^ (not CD4^+^ CD25^+^Treg cells) enhanced CD4^+^CD54RO^hi+^ apoptosis. Cell contact with CD8^+^CD25^+^Treg cells expressing increased granzyme B may induce cell apoptosis of CD4^+^CD45 RO^+^ memory T cells during IVMP pulse therapy. In support of our findings, functional study in autoimmune hepatitis subjects [42] revealed CD4^+^CD25^hi+^ Tregs via direct contact with target cells by modifying levels of regulatory cytokines but not by inducing target cell apoptosis.

Human purified CD4^+^CD25^+^ Treg cells isolated from PBMCs of control and cancer patients suppressed proliferation but did not mediate autologous CD4^+^CD25^−^ responder cell apoptosis [43]. In CD8^+^ knockout recipient mice, transferred Treg cells restored airway inflammation following allergen exposure [44]. We proved that CD4^+^CD25^+^Treg cells require FoxP3^+^-expressing CD8 cells, induced by tolerogenic peptide to suppress lupus activity [45]. This phenomenon arose in our histopathological findings: both CD4^+^FoxP3^+^ and CD8^+^FoxP3^+^Tregs significantly increased in renal tissue of active Class III/IV LN after IVMP pulse therapy. We noted FoxP3 knockdown suppressing granzyme B protein expression in CD8^+^CD25^+^Treg cells. Foxp3+Tregs may suppress immune response by directly killing effector cells; release of granzyme B is implicated in killing effector T and B cells [46]. Our prior study showed that cytotoxic CD8+ Treg cells increasing expression of granzyme B may induce apoptosis of CD4+CD45RO+ memory T cells during allergy immunotherapy [7]. Treg cells can utilize granzyme B to suppress immune responses against tumors and elevated granzyme B(+)/Foxp3(+) cell ratio in tumor cells had a better outcome [47], [48]

In sum, number of CD4^+^CD25^+^FoxP3^+^ and CD8^+^CD25^+^FoxP3^+^ Treg cells in peripheral blood of LN is definitely lower than in healthy controls. Both CD4^+^FoxP3^+^ and CD8^+^FoxP3^+^ Treg cells increased significantly in PBMCs and renal tissue of active class III/IV LN after IVMP pulse therapy. Data support a crucial role of IVMP pulse therapy in suppressing lupus autoimmunity by increasing CD8^+^CD25^+^Treg cells to lessen T cell proliferation, Th1 response in CD4^+^T and increase CD4^+^CD45 RO^+^ apoptosis; together they reduce renal inflammation. We proved IVMP pulse therapy ameliorating autoreactive T cell-mediated auto-immune response by boosting CD8^+^ CD25^+^Treg function.

## Materials and Methods

### Ethics Statement

Criteria for exclusion were previous cyclophosphamide or mycophendate motifil, calcineurin inhibitors, or rituximab. Clinical data including serum creatinine (Cr), glomerular filtration rate (GFR), 24-hour (hr) proteinuria were recorded. Disease activity was assessed by a SLE Disease Activity Index (SLEDAI) score -2k (SLEDAI-2k), validated for use in children [29], [41]. LN activity was defined by renal score of SLEDAI-2k [49], [50]. Patients with SLEDAI <3 were considered inactive (remission), those with SLEDAI ≧8 active [51]. Intervention strategy was based on IVMP pulse therapy and prednisolone. IVMP group patients all received pulse therapy (15–20 mg/kg and maximum with 1 g/day, for 5 days) followed by oral prednisolone 1 mg/kg of body weight and mycophenolate mofetil (cellcept) 280 mg/m^2^ of body surface area twice a day. Doses could be tapered off for proteinuria <1 g/d. Oral prednisolone dosage was tapered to 0.75 mg/kg during the second week, to 0.5 mg/kg during the third. Patients took this dose with subsequent tapering during follow-up as clinically allowed. All subjects provided written informed consent by patients and parents prior to inclusion. The study was approved by the Institutional Review Board of China Medical University Hospital (DMR97-IRB-259).

### Design and Participants

From April 2009 to April 2011, 40 active LN patients (Class III or IV) with heavy proteinuria, age 12 to 18 years (15.2±3.2 years), female/male: 32/8, fulfilling the American College of Rheumatology criteria with disease onset before 16 years of age and nephrotic range proteinuria (>40 mg/m^2^ per hour or >1 g/day per m^2^) were recruited, as detailed in Table 1. Class III or IV LN was classified according to the International Society of Nephrology and Renal Pathology Society [52]. A total of 12 LN patients had no treatment before because of first diagnosis at study entry, and 28 with heavy proteinuria were in relapse at time of the study, median duration of disease was 44 (range 14–112) months.

Peripheral blood mononuclear cells (PBMC) were obtained from LN before (Day 0) and after (Day 6) IVMP pulse therapy. Follow-up serum C3, C4, anti-dsDNA Ab levels and 24 hours daily urine protein were calculated two weeks after IVMP pulse therapy. Serum C3 and C4 levels were determined by nephelometry, serum IgG anti-dsDNA Ab by fluorescence enzyme immunoassay (Pharmacia, Uppsah, Sweden). Plasma samples were evaluated by commercially ELISA kits by assay procedure for the presence of anti-C1q antibody (Orgentec Diagnostika GmbH, Mainz, Germany), assays performed in duplicate. Ten children with similar age distribution and no history of autoimmune disease undergoing examination for health evaluation were recruited as healthy controls. All patients enrolled in each group completed this study, with approval of the institutional ethical review board.

### Renal evaluation and histopathologic assessment

Renal evaluation at the outset and repeat renal biopsies were performed at 3–6 month follow-up, biopsy samples graded according to the World Health Organization (WHO) classification system [36]. Addition, activity and chronicity indices were determined [52].

### Antibody and Reagents

Anti-human CD3, CD4, CD8, CD25, CD45RO, Foxp3, granzyme B, interleukin (IL)-10 mAbs, and isotype-matched control mAbs conjugated with FITC, PE, ECD and PC5 (anti-human IgG1 PC5 conjugated mAb for CD8 & anti-human IgG1 PE conjugated mAb for Foxp3) were obtained from BD Biosciences (San Diego, CA).

### Cell isolation and cell culture

PBMCs were isolated by Ficoll-paque gradient centrifuge (Pharmacia, Uppsala, Sweden), 1×10^6^ cells were cultured and divided on 96-well culture plates in RPMI-1640 culture medium [7]. In some experiments, CD8^+^ or CD4^+^ cells were depleted directly from PBMCs, using microbeads as per manufacturer's protocol (BD Biosciences, San Diego, CA) [7]. CD8^+^CD25^+^ T cells were isolated by CD8^+^ T cell enrichment kit followed by separation with CD25 microbeads (BD Biosciences). Purity of CD8^+^CD25^+^ T cells population analyzed by flow cytometry exceeded 95%. CD4^+^CD25^hi^ Treg cells from normal subjects were purified by EPICS Altra high-speed cell sorter (Beckman Coulter, Miami, FL) and used as positive controls for intracellular Foxp3 expression [7].

### Flow cytometry

Cells were stained for 30 minutes with fluorescein-conjugated mAbs. CD8^+^CD25^+^ Treg cells were permeated, then stained with PE-conjugated, anti-Foxp3 mAb (BD Biosciences). For intracellular IL-10 and Granzyme B cytokine staining, PBMCs were activated with phorbol meristate acetate (PMA) (10 ng/ml) and ionomycin (1 µg/ml) for the last 5 hours of incubation period and brefeldin A (10 µg/ml) (Sigma-Aldrich, St Louis, MO) was added for final hours of stimulation. Cells were fixed, permeated, and stained via standard procedures (eBioscience, San Diego, CA), then analyzed by FACS scan flow-cytometer (FC500, Beckman Coulter, Fullerton, CA), acquiring 10,000 events [7].

### Cell proliferation assay

PBMCs and CD8^+^ depleted-PBMCs were labeled with 5 µM CFSE (Invitrogen, Carlsbad, CA) for 15 minutes at 37°C. Cells were washed twice and stimulated with either anti-human CD3 mAb (1 µg/ml) for five days. For CFSE-suppression assay, CD8^+^CD25^+^ Tregs were added to culture autologus, CD8-depleted and CFSE-labeled PBMCs at 1∶10 ratio, proliferation of CD4^+^ T cells rated by CFSE fluorescence with flow cytometric analysis [7].

### Detection of IFN-r response in CD4 T cells response to autoepitopes

To assess effect of CD4^+^ or CD8^+^ Treg on IFN-r response of CD4^+^ T cells stimulated with autoepitopes, purified CD4^+^CD25^+^ or CD8^+^CD25^+^ Treg cells were added to autologus CD25^+^-depleted PBMCs with nucleosomal histone peptide epitopes (H3: 115–135, H4: 16–39) or whole nucleosomes in presence of anti-CD3/anti-CD28 for three days and then surface stained with anti-CD4, anti-CD8 and intracellular with IFN-r. IFN-r response index ratio was derived by dividing values for corresponding stain of resting control (without peptide). Viable cells gated for being CD4^+^ or CD8^+^ were analyzed for IFN-r production by flow cytometry [7].

### Peptides

All peptides were synthesized by F-moc chemistry, their purity checked by amino acid analysis, as per manufacturer's protocol (Chiron mimotopes and New England Peptide).

### Detection of CD4^+^CD45RO^+^ T cell apoptosis

Purified CD8^+^CD25^+^ Treg cells isolated from LN patients were added to autologus CD25^+^ depleted-PBMCs from lupus nephritis before IVMP and healthy subjects for five days. Apoptosis rate of CD4^+^CD45RO^+^ T cells was obtained by flow cytometry after labeling DNA strand breaks, using a TUNEL kit (Mebstain kit, Lminy, Frcmceu), data confirmed by Annexin V-PI kit (BD Biosciences) [7].

### siRNA transfection

One pair of small-interfering RNAs (siRNA) was synthesized by Invitrogen Life Technology (Invitrogen, Ltd., Taiwan). Peripheral blood CD8^+^CD25^+^ T cells were isolated and pretreated with nucleosomal histone peptide epitope with IL-2 (10 U/ml) for three days, treated with dexamethasone on the third day, then transfected with FoxP3 siRNA (20 µM) using Lipofectamine PNAiMAX (Invitrogen Life Technology). After 48 hours, proteins were extracted for granzyme B western blot, cellular lysates prepared as described previously [7]. Blots were blocked with 4% BSA for 1 h at 22.2°C and probed with rabbit anti-human Ab against granzyme B (Abcam, Abgent, San Diego, CA) and α-tubulin (Sigma) (1/1000), as visualized by ECL using Kodak X-OMATLS film (Eastman Kodak), quantitative data obtained by computing densitometer and Image Quant software.

### Renal Histopathology

Formalin-fixed sections were stained with hematoxylin and eosin, periodic acid Schiff, periodic acid-silver methenamine, and Masson's trichrome [53]. Pathology of glomerular cross-sections (gcs)/kidney was scored on a four-point scale: 0, normal (35–40 cells/gcs); 1, few lesions with slight proliferative change and mild hypercellularity (41–50 cell/gcs); 2, moderate hypercellularity (51–60 cells/gcs); 3, severe hypercellularity (>60 cell/gcs). Minimal change nephrotic syndrome specimens served as normal controls.

### Double Immunohistochemistry

Formalin-fixed and paraffin-embedded blocked renal biopsy specimens were treated according to protocol described previously [38]. Primary antibodies used were anti-mouse monoclonal Ab. (anti-CD3+Ab, Leica, anti-CD4^+^ Ab, Leica; NCL-CD4-IF6, anti-CD8^+^ Ab, Leica; NCL-CD8-295 Biosystems Newcastle UK) at 1∶50 dilution. Secondary Ab used was rabbit monoclonal anti-FoxP3 Ab (Abcam Cambridge UK; ab54501) at 1∶750 dilution, and anti-granzymeB Ab (Abcam, Cambridge UK, ab 89415) at 1∶500 dilution. The CD3^+^ T, FoxP3^+^Treg, CD4^+^FoxP3^+^ and CD8^+^FoxP3^+^ cell counts were calculated as positive cells per HPF at X400 magnification from 10 randomly chosen fields within the same section of the kidney biopsy specimen from an individual patient [53].

### Statistical Analysis

Statistical analysis was performed with commercially available SPSS 12.0 software (SPSS Inc., Chicago, IL), continuous variables expressed as mean ± standard deviation (SD), intergroup comparison by Student t-test, quantitative PCR data analyzed via non-parametric Wilcoxon paired test. Significant differences were defined as p<0.05, group comparison by one-way ANOVA, p-value<0.05 considered significant.
